# A theoretical analysis of taxonomic binning accuracy

**DOI:** 10.1111/1755-0998.13608

**Published:** 2022-05-04

**Authors:** Bianca De Sanctis, Daniel Money, Mikkel Winther Pedersen, Richard Durbin

**Affiliations:** 1 Department of Zoology, University of Cambridge, Cambridge, UK; 2 Department of Genetics, University of Cambridge, Cambridge, UK; 3 Lundbeck Foundation GeoGenetics Centre, GLOBE Institute, University of Copenhagen, Copenhagen, Denmark

**Keywords:** coalescent theory, environmental DNA, metagenomics, taxonomic binning

## Abstract

Many metagenomic and environmental DNA studies require the taxonomic assignment of individual reads or sequences by aligning reads to a reference database, known as taxonomic binning. When a read aligns to more than one reference sequence, it is often classified based on sequence similarity. This step can assign reads to incorrect taxa, at a rate which depends both on the assignment algorithm and on underlying population genetic and database parameters. In particular, as we move towards using environmental DNA to study eukaryotic taxa subject to regular recombination, we must take into account issues concerning gene tree discordance. Though accuracy is often compared across algorithms using a fixed data set, the relative impact of these population genetic and database parameters on accuracy has not yet been quantified. Here, we develop both a theoretical and simulation framework in the simplified case of two reference species, and compute binning accuracy over a wide range of parameters, including sequence length, species–query divergence time, divergence times of the reference species, reference database completeness, sample age and effective population size. We consider two assignment methods and contextualize our results using parameters from a recent ancient environmental DNA study, comparing them to the commonly used discriminative k-mer-based method Clark (*Current Biology*, **31**, 2021, 2728; *BMC Genomics*, **16**, 2015, 1). Our results quantify the degradation in assignment accuracy as the samples diverge from their closest reference sequence, and with incompleteness of reference sequences. We also provide a framework in which others can compute expected accuracy for their particular method or parameter set. Code is available at https://github.com/bdesanctis/binning-accuracy.

## 1 Introduction

Studying organisms in the environment using the genetic traces they leave behind has become an important tool in ecology, evolution and related fields. Environmental DNA (eDNA) is being increasingly used to explore the organismal assemblages in an environmental sample, from diverse and highly complex samples such as the human gut, soil and sea water. eDNA is also increasingly sequenced from ancient samples, taken from ice cores, lake cores, geological or archaeological sedimentary archives, and has been used to reconstruct past environments and phylogeography (Pedersen et al., 2021; Wang et al., 2021). The obtained results are not only used for reconstructing diversity but also for important decision-making within human health, agriculture, restoration and conservation. Traditionally, these data have been limited to simple taxonomic profiling and abundance estimation, but recently studies have started to perform population genetic analyses such as demography, admixture, sex ratio determination and introgression (Adams et al., 2019; Goricki et al., 2017; Parsons et al., 2018; Sigsgaard et al., 2016, 2019; Stepien et al., 2019).

These population genetic analyses require the assignment of individuals reads to reference taxa, which is known as supervised or taxonomic binning. This process can be confounded by local gene tree variation present in recombining systems, as is the case for most eukaryotic taxa. Since some reads will align to more than one reference sequence, these methods necessarily include a decision step on whether to assign these reads and how. Often this is done based on sequence similarity. For example, one could assign the query read to its ‘closest’ reference sequence by choosing the assignment that minimizes mismatches between the query and the reference taxon, which we call the ‘least-mismatch’ method (de Filippo et al., 2018; Kircher, 2012; Prüfer et al., 2010). A more conservative approach is to only assign the query to a reference taxon when it aligns to a reference sequence with no mismatches, which we call the ‘exact-match’ method (Key et al., 2017; Lammers et al., 2021; Pedersen et al., 2016). However, this latter approach will fail to assign query reads that differ from the reference sequence, rendering downstream population genetic analyses that rely on these differences, such as admixture or demography, ineffective (Kircher, 2012).

Many dedicated algorithms for taxonomic binning exist. MALT or MEGAN (Herbig et al., 2016) competitively maps to a user-defined database then employs a lowest common ancestor algorithm. HOPS uses modified alignment parameters to account for damage in ancient DNA (Hübler et al., 2019). SPARSE aligns to clusters of reference genomes (Zhou et al., 2018). Pathoscope assigns microbial reads and removes reads that are similar to a set of filter genomes (Hong et al., 2014). Clark and Kraken use a discriminative k-mer-based approach to assign reads to a reference database (Ounit et al., 2015; Wood et al., 2019). BLAST is also used in this context, even though it was not originally designed for the purpose of binning (Altschul et al., 1990). However, since the premise of taxonomic binning is straightforward, many studies forgo specialized software and align their query sequences to a reference database themselves, for example using bwa (Li & Durbin, 2009) or bowtie2 (Langmead & Salzberg, 2012), then assign query reads to species using least mismatch or exact match as described above or something similar (Anari, 2020; Feuerborn et al., 2020; de Filippo et al., 2018; Key et al., 2017; Pedersen et al., 2021; Prüfer et al., 2010; Warinner et al., 2017). Common choices of reference database are NCBI GenBank (Clark et al., 2015), Refseq (O’Leary et al., 2015), Ensembl (Howe et al., 2020) or a curated set of reference sequences built to suit the metagenomic data set in consideration.

In any of these methods, a decision may leave some query reads unassigned, such as those that align with no mismatches to multiple reference taxa. Furthermore, in many scenarios population variation may mean that the reference from a more distant taxon is actually more similar in the region of the query read, resulting in an erroneous assignment. The error rate resulting from this is influenced by a number of parameters, including the length of the query sequences, the divergence between the query species and its closest reference species (Prüfer et al., 2010), the divergence between related reference species (Brown et al., 2015), and coverage or completeness of the closest reference species in the database (Warinner et al., 2017). Divergence between the query and the reference sequence will decrease accuracy as reads will be less likely to match the reference sequence of the correct species. Using reference species that are too close to each other can actually lead to a negative effect on binning accuracy (Brown et al., 2015), although this can be overcome by mapping to molecular operational taxonomic units (MOTUs), where a unit is defined based on some fixed sequence similarity or clustering algorithm, or by using a lowest common ancestor approach when reads are assigned to multiple species. An incomplete or low-coverage reference sequence that does not contain every position of the genomes will cause reads to be assigned to the closest related species instead, leading to incorrect assignments (Warinner et al., 2017).

Though it is widely understood that parameters concerning the read, database or species in question can improve or reduce the accuracy of this binning step, recommendations and practices for how to cope with this differ among the literature. For instance, filters or criteria in existing studies can be based on sequence similarity (e.g. 85% in (Krause-Kyora et al., 2018), 95% in (Willerslev et al., 2014) and (Haile et al., 2009), 100% (Pedersen et al., 2016)), alignment score, total percentage of identifiable sequences assigned to that species (e.g. 1% (Slon et al., 2017)), read length, divergence between reference species (e.g. 3% in (Brown et al., 2015)) and more.

**Figure 1:**
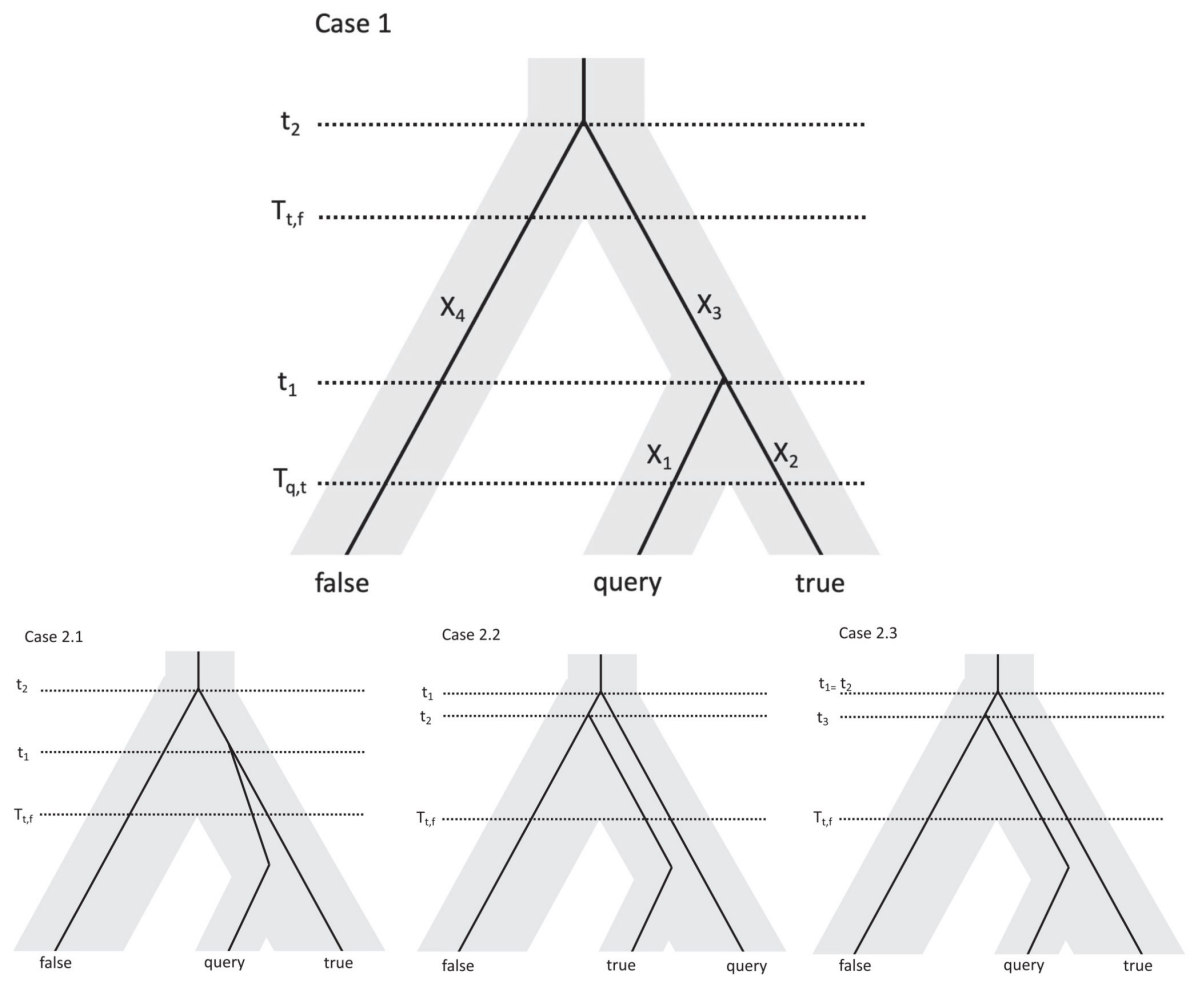
An illustration of the four possible scenarios. Here, *T*_*t,f*_ represents the divergence time between the true and false species, and *T*_*q,t*_ represents the divergence time between the query and true species, the latter of which is irrelevant in the bottom three cases. Note the change in order of the branches in cases 2.2 and 2.3. See Methods for details

To our knowledge, the exact relationships of these parameters to binning accuracy have not been quantified, in either relative or absolute terms (although see (Nielsen & Matz, 2006) for a related study). Because of this, it may be difficult to know which filtering or database construction steps or methods to use and prioritize in practice. Here, we use a two-species coalescent model to quantify the effects of relevant database, population genetic or read parameters on the accuracy of the binning step. We consider a simplified case for which analytical results can be obtained under a coalescent model (Figure 1). We consider three species or populations: one for the ‘query’ species from which the query sequence is sampled, one for the ‘true’ closest species represented in the reference database and one for the ‘false’ species which is the next closest in the reference database. For simplicity, we use the term ‘species’ here in all three cases, but each may equally well be a subspecies, population or other genetically mixing taxonomic group.

Our model includes parameters not usually mentioned in this context, such as the age of the sample, which can impact accuracy when comparing to a present-day reference sequence. In total, we consider how sequence length, species–query divergence time, reference species divergence time, reference species completeness, sample age and effective population size impact the accuracy of the taxonomic identification of a single sequence. Although we use a two-species database and consider only two assignment methods for simplicity and exactness, we believe that the conclusions from our model concerning the relative impact of parameters on accuracy can be applied more generally to inform an understanding of error rates in analyses using larger databases or other binning algorithms. In particular, because the errors arise from genuine overlap between the patterns of similarity of the query to true or false taxa, we expect the relative impact on the error rate of population genetic parameters that affect this overlap to be similar regardless of the algorithm or reference database size.

This problem of assigning individual sequences to taxa is not a problem isolated to the field of environmental DNA, but also appears in ancient DNA studies, where a large fraction of reads collected from a fossil can originate from microbial contamination (de Filippo et al., 2018). However, in ancient DNA studies, even with a high level of expected binning accuracy, additional criteria need to be met to ensure the authenticity of the reads. This includes, for example, comparing edit distance distributions to related species and confirming signs of ancient DNA damage such as deamination, and is covered in depth elsewhere (Orlando et al., 2021; Renaud et al., 2019).

## 3 Materials and Methods

Our analysis will start with the case where the query and true species are the same that is where the query sample derives from the same panmictic population from which the reference sequence for the true species was obtained. We then go on to extend to the case where there was some divergence between the populations from which the query and the true reference sequence were sampled, which is typical in practice. We also first consider the case when all individuals are from the present day and the two references are complete, but expand on all of these assumptions in the following sections. We note that this approach assumes a single reference sequence for each species in the reference database—there are more complex scenarios that use multiple reference sequences within a single species or other taxonomic unit.

From here on, we refer to the query sequence as q, the true sequence as t and the false sequence as f. We show calculations for the least-mismatch method, where the correct assignment probability is the probability that the number of mutations between the query and the false sequence is greater than the number of mutations between the query and the true sequence. Denote the number of mutations between the query and the true sequence as the random variable *K*_*t*_, and the number of mutations between the query and the false sequence as the random variable *K*_*f*_. Notably, these two variables are not independent. We then have in the least-mismatch method *P*(*K*_*t*_ < *K*_*f*_) as the probability of correct assignment, *P*(*K*_*t*_ > *K*_*f*_) as the probability of incorrect assignment and *P*(*K*_*t*_ = *K*_*f*_) as the probability of no assignment. First, we will calculate the probability of correct assignment.

Denote the divergence time of the true and false species as *T*_*t,f*_, and the divergence time of the true and query species as *T*_*q,t*_, both in generations. We first assume that *T*_*q,t*_ = 0 and generalize this below. There are two cases to consider: either the query coalesces with the true sequence before time *T*_*t,f*_, or after. The latter is possible in the case of incomplete lineage sorting and often occurs with negligible probability unless the two reference sequences are closely related. An illustration of the possible coalescent scenarios is shown in Figure 1. For the remainder of this section, we use *T* = *T*_*t,f*_ for ease of reading.

### Case 1

The query coalesces with the true sequence before time T. This happens with probability 1 − e^−*T*/2*N*^, where *N* is the effective population size of the population *q* and *t* are drawn from. In this case, let *t*_1_ < *T* be the coalescent time for *q* and *t*, and let *t*_2_ > *T* be the coalescent time for q and f. Let *X*_1_ and *X*_2_ be the number of mutations on the branches from *q* and *t*, respectively, to the common ancestor of *q* and *t, X*_3_ the number of mutations on the branch between the common ancestor of *q* and *t* to the root (the most recent common ancestor of *q, t* and *f*) and *X*_4_ be the number of mutations on the branch between *f* and the root. This scenario is shown in the top of Figure 1, with *T*_*q,t*_ = 0 (the generalization for *T*_*q,t*_ ≠ 0 is derived below). We want to compute

$$\begin{array}{c} P\left(K_{t}<K_{f}\right)\;=P\left(X_{1}+X_{2}<X_{1}+X_{3}+X_{4}\right)=P\left(X_{2}<X_{3}+X_{4}\right)\\ =P\left(Pois\left(\mu t_{1}\right)<Pois\left(\mu \left(t_{2}-t_{1}\right)\right)\right) \end{array}$$

given that *t*_1_ < *T* ≤ *t*_2_, since we expect that the number of mutations on each branch is Poisson distributed. Let *A* = Pois (*µ* (2*t*_2_ − *t*_1_)) and *B* = Pois (µ*t*_1_) where *μ* is the mutation rate of the sequence per generation, the product of the per base mutation rate and the match length. Then, given *t*_1_ and *t*_2_, we want

$$\begin{array}{c} \;P(B<A)=P(A>B)=\sum_{k=0}^{\infty }P(A>B;B=k)P(B=k)=\sum_{k=0}^{\infty }P(A>k)P(B=k)\\ \;=\sum_{k=0}^{\infty }\left(\sum_{I=k+1}^{\infty }\frac{\lambda_{A}^{l}e^{-\lambda_{A}}}{I!}\right)\frac{\lambda_{B}^{k}e^{-\lambda_{B}}}{k!}=\sum_{k=0}^{\infty }\left(\sum_{I=k+1}^{\infty }\frac{{(\mu \left(2t_{2}-t_{1}\right))}^{l}e^{-\mu \left(2t_{2}-t_{1}\right)}}{I!}\right)\frac{{\left(\mu t_{1}\right)}^{k}e^{-\mu t_{1}}}{k!}\\ =\sum_{k=0}^{\infty }\sum_{I=k+1}^{\infty }\frac{{(\mu \left(2t_{2}-t_{1}\right))}^{I}{\left(\mu t_{1}\right)}^{k}e^{-\mu \left(2t_{2}\right)}}{I!k!} \end{array}$$

Since *t*_1_ and *t*_2_ are unknown, we take the expectation over *t*_1_ and *t*_2_ given *t*_1_ < *T* ≤ *t*_2_. That means, in the case that the query coalesces with the true sequence before time T, the probability of correct assignment as a function of *T, μ* and *N* is

$$\begin{array}{c} P_{CA}\left(T,\mu ,N;t_{1}<T<t_{2}\right)\\ \;=\frac{\int_{T}^{\infty }\int_{0}^{T}\frac{{\text{e}}^{-t_{1}/2N}}{2N}\frac{{\text{e}}^{-t_{2}/2N}}{2N}\left({\text{Σ}}_{k=0}^{\infty }{\text{Σ}}_{l=k+1}^{\infty }\frac{{(\mu \left(2t_{2}-t_{1}\right))}^{\prime }{\left(\mu t_{1}\right)}^{k}{\text{e}}^{-\mu \left(2t_{2}\right)}}{l!k!}\right)\text{d}t_{1}\text{d}t_{2}}{\left({\text{e}}^{-T/2N}\left(1-{\text{e}}^{-T/2N}\right)\right)} \end{array}$$

where the first two exponentials are for the distributions of *t*_1_ and *t*_2,_ respectively, and where it is assumed that the effective sizes of the population consisting of *q* and *t* before time *T* and the ancestral population to *q, t* and *f* after time *T* are equal and of value *N*. The denominator is obtained by integrating the numerator without the double summation inside and acts as a normalizing constant.

### Case 2

The query does not coalesce with the true sequence before time *T*. This happens with probability e^−*T*/2*N*^. In this case, we essentially have 3 sequences at time *T*, which have not coalesced with each other, and therefore three subcases depending on which two coalesce first, each of which will happen with probability 1/3.

#### Case 2.1

*T* ≤ *t*_1_ < *t*_2_, so that the query and true sequence coalesce first (but after time *T*). In this case, we can make a similar argument to Case 1 and simply have to change the denominator to the relevant domain, and the limits on the integral, to get

$$=\frac{\begin{array}{l} \;P_{CA}\left(T,\mu ,N;T\leq t_{1}<t_{2}\right)\\ \int_{T}^{\infty }\int_{t_{1}}^{\infty }\frac{e^{-t_{1}/2N}}{2N}\frac{e^{-t_{2}/2N}}{2N}\left({\text{Σ}}_{k=0}^{\infty }{\text{Σ}}_{l=k+1}^{\infty }\frac{{(\mu \left(2t_{2}-t_{1}\right))}^{\prime }{\left(\mu t_{1}\right)}^{k}e^{-\mu \left(2t_{2}\right)}}{l!k!}\right)\text{d}t_{2}\text{d}t_{1} \end{array}}{\left(\frac{1}{2}e^{-T/N}\right)}$$

Again, the denominator here is obtained by integrating the numerator without the double summation inside.

#### Case 2.2

*T* ≤ *t*_2_ < *t*_1_, so that the true and false sequence coalesce first. In this case, the phylogenetic distance between the query and the true sequence is the same as the phylogenetic distance between the query and the false sequence. Since the mutations on the query branch will be the same in both cases, we will have *P*(*K*_*t*_ < *K*_*f*_) simply when the number of mutations on the true branch is less than the number of mutations on the false branch. Both of these branches have length *t*_2_, so we would like the probability that one Poisson process with mean *μt*_2_ is bigger than another which is identically and independently distributed. Given *t*_2_, this will be

$$\sum_{k=0}^{\infty }\left(\sum_{l=k+1}^{\infty }\frac{{\left(\mu t_{2}\right)}^{l}{\text{e}}^{-\mu t_{2}}}{l!}\right)\frac{{\left(\mu t_{2}\right)}^{k}{\text{e}}^{-\mu t_{2}}}{k!}=\sum_{k=0}^{\infty }\sum_{l=k+1}^{\infty }\frac{{\left(\mu t_{2}\right)}^{l+k}{\text{e}}^{-2\mu t_{2}}}{l!k!}$$

And now, as usual, we integrate over the relevant domain to get

$$\begin{array}{c} P_{CA}\left(T,\mu ,N;T\leq t_{2}<t_{1}\right)\\ \;=\int_{T}^{\infty }\int_{t_{2}}^{\infty }\frac{e^{-t_{1}/2N}}{2N}\frac{e^{-t_{2}/2N}}{2N}\sum_{k=0}^{\infty }\sum_{l=k+1}^{\infty }\frac{{\left(\mu t_{2}\right)}^{l+k}e^{-2\mu t_{2}}}{l!k!}\text{d}t_{1}\text{d}t_{2}/\left(\frac{1}{2}e^{-T/N}\right) \end{array}$$

#### Case 2.3

*t*_2_ = *t*_1_ > *T*, so that the query and the false sequence coalesce first. This is possible due to incomplete lineage sorting, but only if it happened at a point older than *T*. Let *t*_3_ be the time at which the query and the false sequence coalesce. We want the probability that a Poisson process with mean μ(2*t*_1_−*t*_3_) is less than one with mean *μt*_3_, which for a given *t*_1_ and *t*_3_ is

$$\begin{array}{c} \;\sum_{k=0}^{\infty }\left(\sum_{l=k+1}^{\infty }\frac{{\left(\mu t_{3}\right)}^{l}{\text{e}}^{-\mu t_{3}}}{I!}\right)\frac{{(\mu \left(2t_{1}-t_{3}\right))}^{k}{\text{e}}^{-\mu \left(2t_{1}-t_{3}\right)}}{k!}\\ =\sum_{k=0}^{\infty }\sum_{l=k+1}^{\infty }\frac{{\left(\mu t_{3}\right)}^{l}{(\mu \left(2t_{1}-t_{3}\right))}^{k}{\text{e}}^{-\mu 2t_{1}}}{l!k!} \end{array}$$

Integrating over the relevant domain gives

$$\frac{\begin{array}{l} \;P_{CA}\left(T,\mu ,N;t_{2}=t_{1}<T\right)=\\ \int_{T}^{\infty }\int_{t_{3}}^{\infty }\frac{{\text{e}}^{-t_{1}/2N}}{2N}\frac{e^{-t_{3}/2N}}{2N}\sum_{k=0}^{\infty }\sum_{l=k+1}^{\infty }\frac{{\left(\mu t_{3}\right)}^{\prime }{(\mu \left(2t_{1}-t_{3}\right))}^{k}{\text{e}}^{-\mu 2t_{1}}}{l!k!}\text{d}t_{1}\text{d}t_{3} \end{array}}{\left(\frac{1}{2}{\text{e}}^{-T/N}\right)}$$

In summary, the probability of correctly assigning the query sequence to the true sequence is

$$\begin{array}{c} P_{CA}(T,\mu ,N)=\left(1-e^{-T/2N}\right)P_{CA}\left(T,\mu ,N;t_{1}<T<t_{2}\right)+\\ \;\frac{e^{-T/2N}}{3}(P_{CA}\left(T,\mu ,N;T\leq t_{1}<t_{2}\right)+P_{CA}\left(T,\mu ,N;T\leq t_{2}<t_{1}\right)\\ +P_{CA}\left(T,\mu ,N;t_{2}=t_{1}<T\right)) \end{array}$$

However, in many cases *T*/2*N* ≫ 1 so that the latter three terms are negligible and we can obtain a sufficiently good approximation by only calculating the first term.

**FIgure 2:**
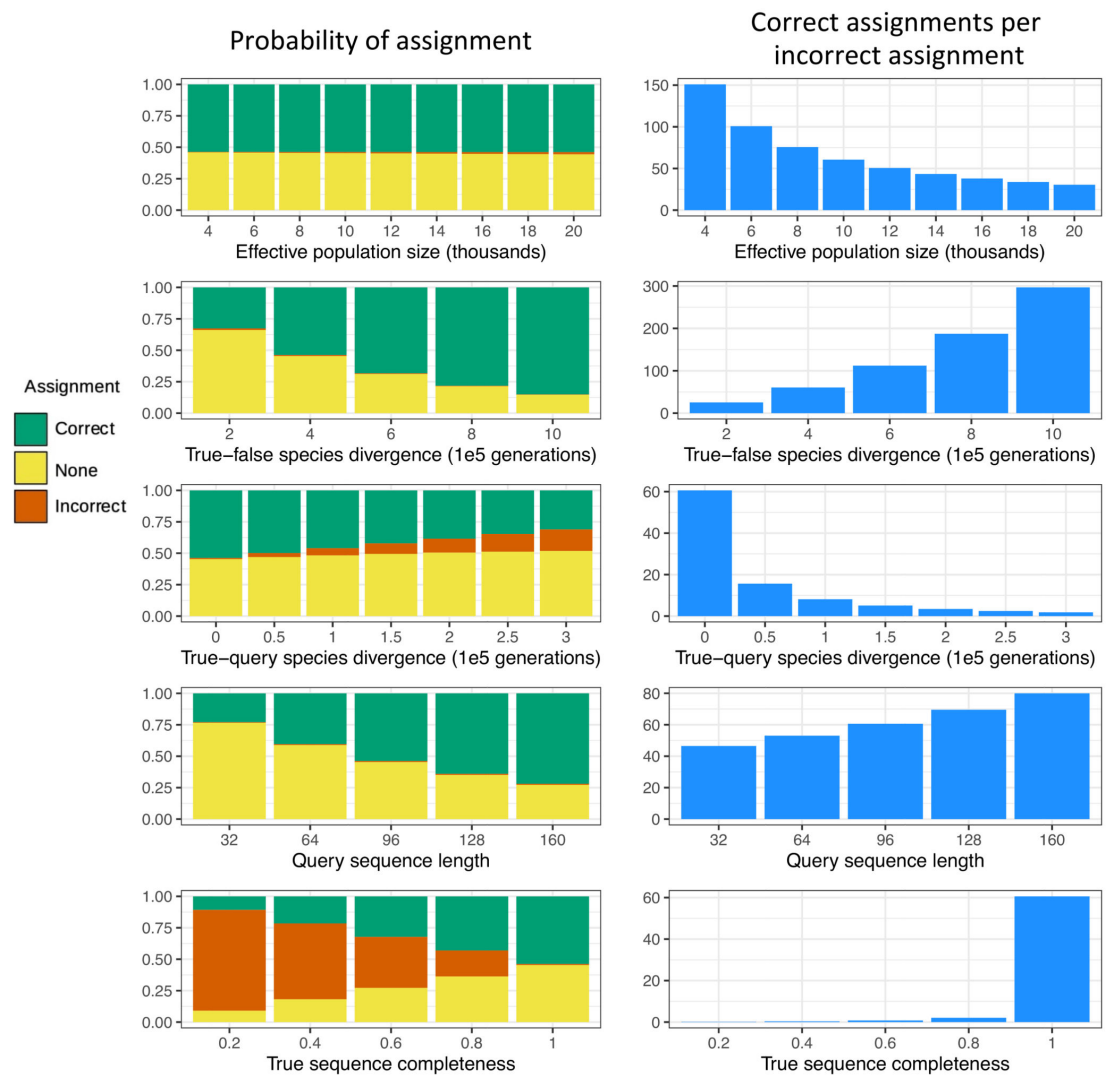
Left: Probability of assigning the query sequence correctly (to the true reference sequence) in green, of assigning the query sequence incorrectly (to the false reference sequence) in red, and of making no assignment in yellow, using the least-mismatch method. Right: The expected number of correct assignments (to the true reference sequence) made for every one incorrect assignment (to the false reference sequence). In both, each row varies a different parameter while keeping the others constant

Similar calculations give the results for the ‘exact-match’ method, the probability of incorrect assignment, and the probability of not making an assignment. The latter occurs when a query matches both reference sequences equally well in the ‘least-mismatch’ method, or when it matches both equally well or does not match either with zero mismatches in the ‘exact-match’ case. We compute the probabilities of correct, incorrect and no assignment, while varying the input parameters and assignment method, as shown in Figure 2 and Figure S1. For very high population sizes such as in Figure S1, terms in the integrand can become small enough to be prone to errors, and so we instead scaled time appropriately to compute the equivalent probabilities with smaller population sizes (i.e. divided the effective population size, multiplied the mutation rate and divided all divergence times by a fixed scaling factor). Code is available at https://github.com/bdesanctis/binning-accuracy.

### 2.1 Extensions to the model

Next, we can generalize this to include ages of each of the sequences if they are not contemporaneous, and to include a nonzero divergence time between the query and true species. The latter is difficult to escape at a small scale, because in most applications the query will not be expected to perfectly derive from the true reference species (in the coalescent sense we are using here), but as we shall see, a small level of divergence will not significantly impact the assignment probability. However, this can also present itself on a larger scale when the query species is not in the reference panel at all, but assignments are made to the closest relevant species. This applies especially when the query represents a new, extinct or previously unsequenced species.

Let *T*_*q,t*_ denote the coalescence time between the true and query species or populations. For clarity, we will rename the coalescence time of *t* and *f* species, previously called *T* in the above section, to *T*_*t,f*_. We then require *T*_*q,f*_ < *T*_*t,f*_. Times which are variables, will remain denoted by lowercase *t*s, with appropriate subscripts.

Denote the age of each of the individuals as *A*_*q*_, *A*_*t*_ and *A*_*f*_, measured in generations. There are constraints on these ages with regard to the coalescence times for consistency. That is, we require *A*_*q*_, *A*_*t*_ < *T*_*q,t*_ and *A*_*f*_, *A*_*t*_ <*T*_*t,f*_.

Incorporating these two generalizations into each case is fairly straightforward. In case 2, T_q,t_ is irrelevant, and so generalizing these is a simple matter of building in ages. This gives

$$\begin{array}{c} P_{CA}\left(T_{tf},T_{q,\text{t}},\mu ,N,A_{q},A_{t},A_{f};t_{1}<T_{t,f}<t_{2}\right)=\\ \;\frac{\int_{T_{tf}}^{\infty }\int_{T_{q,t}}^{T_{tf}}\frac{{\text{e}}^{-t_{1}/2N}}{2N}\frac{{\text{e}}^{-t_{2}/2N}}{2N}\left(\sum_{k=0}^{\infty }\sum_{l=k+1}^{\infty }\frac{\mu^{l+k}{\left(2t_{2}-t_{1}-A_{f}\right)}^{\prime }{\left(t_{1}-A_{t}\right)}^{k}{\text{e}}^{-\mu \left(2t_{2}-A_{f}-A_{t}\right)}}{1!\text{k}!}\right)\text{d}t_{1}\text{d}t_{2}}{\left({\text{e}}^{-T_{tf}/2N}\left({\text{e}}^{-T_{q,t}/2N}-{\text{e}}^{-T_{tf}/2N}\right)\right)} \end{array}$$

$$\begin{array}{c} \;P_{CA}\left(T_{t,f},T_{q,t},\mu ,N,A_{q},A_{t},A_{f};T_{t,f}\leq t_{1}<t_{2}\right)=\\ \frac{\int_{T_{tf}}^{\infty }\int_{t_{1}}^{\infty }\frac{e^{-t_{1}/2N}}{2N}\frac{e^{-t_{2}/2N}}{2N}\left(\sum_{k=0}^{\infty }\sum_{l=k+1}^{\infty }\frac{\mu^{\prime +k}{\left(2t_{2}-t_{1}-A_{f}\right)}^{\prime }{\left(t_{1}-A_{t}\right)}^{k}e^{-\mu \left(2t_{2}-A_{f}-A_{t}\right)}}{1!k!}\right)\text{d}t_{2}{\text{d}}_{1}}{\left(\frac{1}{2}{\text{e}}^{-T_{tf}/N}\right)} \end{array}$$

$$\begin{array}{c} P_{CA}\left(T_{t,f},T_{q,t},\mu ,N,A_{q},A_{t},A_{f};T_{t,f}\leq t_{2}<t_{1}\right)=\\ \;\int_{T_{tf}}^{\infty }\int_{t_{2}}^{\infty }\frac{{\text{e}}^{-t_{1}/2N}}{2N}\frac{{\text{e}}^{-t_{2}/2N}}{2N}\underset{k=0}{\sum }\underset{l=k+1}{\sum }\frac{\mu^{l+k}{\left(t_{2}-A_{f}\right)}^{\prime }{\left(t_{2}-A_{t}\right)}^{k}{\text{e}}^{-\mu \left(2t_{2}-A_{t}-A_{t}\right)}}{l!k!}\text{d}t_{1}\text{d}t_{2}/\left(\frac{1}{2}{\text{e}}^{-T_{tf}/N}\right) \end{array}$$

$$\begin{array}{c} P_{CA}\left(T_{tf},T_{q.t},\mu ,N,A_{q},A_{t},A_{f};t_{2}=t_{1}<T_{t,f}\right)\\ \;\int_{T_{tf}}^{\infty }\int_{t_{3}}^{\infty }\frac{{\text{e}}^{-t_{1}/2N}}{2N}\frac{{\text{e}}^{-t_{3}/2N}}{2N}\underset{k=0}{\sum }\underset{l=k+1}{\sum }\frac{\mu^{l+k}{\left(t_{3}-A_{f}\right)}^{\prime }{\left(2t_{1}-t_{3}-A_{t}\right)}^{k}{\text{e}}^{-\mu \left(2t_{1}-A_{t}-A_{f}\right)}}{l!k!}dt_{1}\text{d}t_{3}/\left(\frac{1}{2}{\text{e}}^{-T_{tt}/N}\right) \end{array}$$

The probability of each of these cases differs with ancient samples as well. In particular, the probability of the true and query individuals coalescing before *T*_*t,f*_ is

$$\text{exp}\left(-\text{min}\left(\frac{\left(T_{t,f}-A_{q},T_{t,f}-A_{t}\right)}{2N}\right)\right)$$

and the other probabilities are modified similarly.

### 2.2 Modelling incomplete reference sequences

So far, we have assumed completeness of both reference genomes, where we can define completeness as the fraction of covered bases. Note that the fraction of covered bases ranges from 0 to 1 and is not equivalent to coverage. In particular, we are not considering base quality or depth here, only whether or not the bases are represented in the reference sequence. This concept of completeness is sometimes referred to as breadth of coverage.

In the case of an incomplete reference sequence, there will be regions of the reference genomes to which the query will not align due to its lack of representation in the reference, which will happen with probability directly proportional to its completeness. Let *C*_*t*_ and *C*_*f*_ denote the completeness of the true and false sequences, respectively. We have three relevant cases:

- The query read aligns to both true and false sequences, with probability *C*_*t*_ *C*_*f*_ / (1 − (1 − *C*_*t*_) (1 − *C*_*f*_))
- The query read aligns to the true sequence but not the false sequence, with probability *C*_*t*_ (1 − *C*_*f*_ / (1 − (1 − *C*_*t*_ 1 − *C*_*f*_))
- The query read aligns to the false sequence but not the true sequence, with probability (1 − *C*_*t*_) *C*_*f*_ / (1 − (1 − *C*_*t*_) (1 − *C*_*f*_))

where the denominator is there in every case to represent the condition that the query does align to at least one of the two references. In the first case, the correct assignment probability is that presented above as *P*_*CA*_ (*T, µ, N*). In the second, the correct assignment probability is 1, and in the third, it is 0. Therefore, accounting for reference completeness, the probability of assigning the query sequence to the true sequence is

(1) $$\frac{C_{t}C_{f}P_{CA}(T,\mu ,n)+C_{t}\left(1-C_{f}\right)}{1-\left(1-C_{t}\right)\left(1-C_{f}\right)}$$

### 2.3 Simulations

Next, we wrote a simulation script using Python with msprime (Kelleher et al., 2016) as an engine. The script takes as input the genome length, effective population size, number of reads, read length, mutation rate, true–false divergence time, true–query divergence time, generation length, ages of the samples and recombination rate. We build a phylogenetic tree using the input parameters, pass the relevant parameters and tree to msprime to create variable sites between the three sequences, randomly simulate nucleotides to fill in the remaining nonvariable sites and obtain diploid sequences for each of the true, query and false sequences. We randomly choose one of the two strands to represent the true and false sequences, and sample reads with equal probability from both strands of the query sequence. We can then either output these sequences and reads as fasta files, or directly compare the number of mismatches with the query reads to the true and false sequences to obtain assignment probabilities under each method. We note that msprime throws an error if one attempt to build a tree with a length zero branch, so we used *T*_*q,t*_ = 0.0001 in the simulations to compare with the theoretical results for *T*_*q,t*_ = 0. To validate our theoretical predictions, we simulated all values in Figure 2, as shown in Table S1. The simulation script is available at https://github.com/bdesanctis/binning-accuracy.

Next, we wanted to compare the least-mismatch and exactmatch methods to a commonly used existing taxonomic binning method, and to do this based on a context previously established by an empirical study (Pedersen et al., 2021). For the former, we used a method based on discriminative k-mers, called Clark (Ounit et al., 2015), and ran it with default settings on the simulated fasta sequences and reads. The parameter set used was that from a recent ancient environmental DNA study which contained two Ursid species, American black bear *Ursus americanus* and the extinct giant short-faced bear *Arctodus simus*, which diverged approximately at *T*_*q,t*_ = 13.4 mya (Pedersen et al., 2021). In the study, they used a spectacled bear *Tremarctos ornatus* reference sequence to assign giant short-faced bear reads. This means the true and the query sequence were from different species, diverged approximately *T*_*q,t*_ = 5 mya (Pedersen et al., 2021). As in the original study, we also used an Ursid generation length of 6 years, an effective population size of 10,000, a mutation rate of 0.6e−8 per site per generation, a query age of 14 kya and an age of 0 for the true and false sequences. We further used a recombination rate of 1e−8 per site and read lengths of 40 and 100 bp. To obtain accuracy estimates, we simulated 10,000 reads and a genome length of 10 million, and mapped the query reads back to the true and false sequences using bwa aln (Li & Durbin, 2009) with default parameters, then counted the mismatches between each read and the reference sequences using a custom script and samtools (Li et al., 2009). In the least-mismatch method, if a read did not align to one of the reference sequences at all, we assigned it to the other.

True sequence completeness True sequence completeness Lastly, we wanted to test the effect of deamination, a damage process that occurs in ancient DNA and appears as C to T or G to A transition SNPs in the reads. Effectively, this places ‘mutations’ on the query branch (see Figure 1), and therefore, in theory, it should decrease both correct and incorrect assignments of the exact-match method, and not significantly impact the least-mismatch method. We used gargammel (Renaud et al., 2017) to add deamination to our simulated query reads, using a misincorporation matrix from the original data set from the sample library ‘UE1210 Mex 18 Lib4’. We then re-ran the above analysis on the deaminated reads.

## 3 Results and Discussion

### 3.1 Theoretical results

We first calculate the theoretical probability of a single query sequence being assigned to either the correct reference sequence, the incorrect reference sequence or neither sequence under a coalescent model. In particular, we model the situation where a single read or query sequence aligns to two reference sequences, and (a) the query is assigned to the reference sequence to which it has the least number of mismatches (referred to as ‘least mismatch’), or (b) the query is assigned to a reference sequence if they have no mismatches, and this is not the case for any other reference sequence (referred to as ‘exact match’). If the query sequence has the same number of mismatches as both reference sequences in either case, or does not exactly match any reference sequence in the exactmatch case, it is not assigned to either.

The relevant theory, which is detailed in the Methods section, relies on a few key parameters. First, we need to differentiate between the coalescence time of the true, false and query sequences, and the divergence times of their respective species or populations (Figure 1). We use the terms ‘true–query species divergence time’ and ‘true–false species divergence time’ to refer to those of the latter class, as the exact coalescence times of the true, false and query sequences will be unknown in practice (see Methods and Figure 1 for details). We also incorporate the length of the query sequence, the mutation rate, the effective population size (which is assumed to be constant) and the completeness of the reference sequences, the latter of which is defined as the proportion of sites represented in the reference genome. Further generalizations which incorporate the age of each of the sequences are derived in the Methods section. We use a fixed set of parameters and modify one at a time over a range to determine the relative impact of different parameters on assignment accuracy.

In Figure 2, we used a baseline parameter set consisting of the following parameters: the query sequence length *k* = 96, a mutation rate of *μ* = *k*·1e−8 per generation, an effective population size of *N*_*e*_ = 10,000, a query–true species divergence time of zero generations (i.e. assuming they come from the same population), a true—false species divergence time of 400,000 generations, and a true sequence completeness and a false sequence completeness of 1. On each row of Figure 2, we use this baseline parameter set and modify one parameter at a time. On the left of Figure 2, we plot the probability of correctly assigning the query sequence to the true sequence in green, the probability of incorrectly assigning it to the false sequence in red, and the probability of not assigning it to either sequence in yellow. Since many of the incorrect assignment probabilities are too small to visually compare in this way, we also show, on the right, the number of expected correct assignments per one incorrect assignment. All results shown in Figure 2 are for the least-mismatch assignment method. The exact-match assignment method gives similar results in general, and the differences between the two are small for the parameters shown in Figure 2, with exact values given in Table S1. Our baseline parameter set leads to an expected 60.6 correct assignments for every incorrect assignment using the least-mismatch method.

We validated these theoretical results using simulations as described in Methods (see Table S1), where we also give results for the exact-match assignment method. For each parameter combination, we simulated 10,000 query reads from a sequence of length 10 million with a recombination rate of 1e−8, so that each query read effectively acted as an independent replicate. To check that our simulated values matched our theoretical values, we performed two-sided binomial tests for each parameter combination, with p-values shown in Table S1. Only five were significant at a level of *p* =.05, which is consistent with random chance since we checked a total of 128 different parameter combinations.

As seen in the first row of Figure 2, a smaller effective population size will lead to a more recent coalescence of the query and true sequence, so that there will be fewer expected differences between the two sequences and we have a higher chance of correctly assigning the query based on these differences. We note that selection can reduce the effective population size locally in the genome, with similar consequences (Liu & Mittler, 2008). We would also expect a higher correct assignment probability if the true and false species diverged a long time ago, as shown in the second row, in which case there will be more differences between the true and false sequences, and therefore more between the query and false sequences. Adaptive selection can also locally increase the number of differences. However, though both these factors impact the number of expected correct assignments per incorrect assignments, the effective population size barely impacts how many total sequences one expects to assign, whereas a higher true–false divergence time or local adaptive selection will lead to more total assigned sequences.

Next, if we assume the query and the true sequence come from different populations or even different species (Figure 2 row 3), the probability of correct assignment can drop dramatically. Even when these two populations diverged 50,000 generations ago, compared to the 400,000 generation divergence of the true and false species, we would expect less than 20 correct assignments per incorrect assignment. A higher query–true divergence time amplifies the problem quickly, emphasizing the importance of using reference sequences, which are as close as possible to the expected query species. In practice, we might expect a small but nonzero true–query divergence time even when using a reference sequence assumed to be very similar to the query, since in most cases one cannot assume that the query sequence and the reference sequence are from the same population. Furthermore, when using an ancient query sequence, we would expect the populations to have diverged some time ago, leading to a higher query–true divergence time and higher expected error rates. Another relevant case is when an ancient query sequence belongs to an extinct species for which there is no reference genome, and therefore, the sequence of a nearby species must be used in the reference set, leading to a high true–query divergence time and consequently increasing error rates. The age of a sample affects assignment accuracy in the exact same way as the query–true divergence time, and so is not shown here, though the relevant equations are given in the Methods.

The length of the query sequence has some impact on the assignment accuracy, where higher sequence lengths correspond to higher accuracies. Often a 30-bp minimum sequence length is emphasized (Schubert et al., 2012), and while this is helpful, other parameters have a far greater effect on the assignment accuracy.

By far, the parameter that has the most impact on the assignment accuracy in this parameter range is the completeness of the true sequence, where completeness is defined as the fraction of represented sites in the reference genome. As can be seen in the last row of Figure 2, failing to have even a fifth of the sites in the reference genome covered will lead to a significant assignment error. This is a natural consequence of Equation 1. Perhaps the most important consideration to maximize binning accuracy is therefore ensuring that one uses high-quality and complete reference genomes in the reference database. However, when this is not possible, a practical fix could be to remove from consideration those genomic regions, and associated query reads, which are not represented in all of the reference genomes where one would expect them to be. In particular, the query reads most susceptible to incorrect assignment due to insufficient reference genome completeness are those which map to a single region in one of the reference genomes, but fail to map to the other due to the lack of representation of that region in that reference genome. In practice, identifying these regions would probably require aligning the reference sequences.

Having multiple nearby reference sequences is not modelled here, but if they spanned the population to which the query belongs or has recently diverged from, this would also likely reduce assignment errors by increasing the chance of the query coalescing more quickly with one of these sequences. We are also not considering the case where we have high read coverage, where it may be more appropriate to undertake metagenomic assembly and assign contigs (Yang et al., 2021).

Microbial populations can have much larger effective population sizes than those shown in Figure 2. Because of this, in Figure S1, we also show theoretical results for effective population sizes two orders of magnitude larger than those in Figure 2, in the range of *N*_*e*_ = 1,000,000, with all other parameters as in Figure 2. The general patterns described above persist, but there is a much higher incorrect assignment probability, and the ratio of correct to incorrect assignments expected is often close to 1. This means that, with very high effective population sizes, individual read or sequence assignments of this type can be extremely unreliable, and one may have a high proportion of their reads incorrectly assigned.

### 3.2 Simulation results

We next wanted to (a) show how our two assignment methods compared with a commonly used binning software, (b) present results in a context previously established by an empirical study and (c) study how deamination, an ancient DNA damage process, might affect accuracy. We therefore compared the least-mismatch and the exact-match methods to Clark (Ounit et al., 2015), a discriminative k-mer-based method often used for taxonomic binning, on a parameter set motivated by a recent ancient environmental DNA study (Pedersen et al., 2021). This study contained reads from two separate bear species: the American black bear *Ursus americanus* and the extinct giant short-faced bear *Arctodus simus*. The closest living relative to the extinct *Arctodus* is the spectacled bear *Tremarctos ornatus*. Here, we simulated *Arctodus simus* query reads and consider the accuracy when attempting to assign these reads to the closest ‘true’ reference sequence *Tremarctos ornatus*, with the ‘false’ reference sequence as *Ursus americanus*. The *Ursus–Arctodus* divergence time is approximately *T*_*t,f*_ = 13.4 million years ago (Pedersen et al., 2021), the *Arctodus–Tremarctos* divergence time is approximately *T*_*t,f*_ = 5 million years ago, and other relevant parameters are given in the Methods section. We simulated query reads from *Arctodus simus* using read lengths of *k* = 40 and *k* = 100, both with and without deamination, a type of ancient DNA damage.

**Figure 3:**
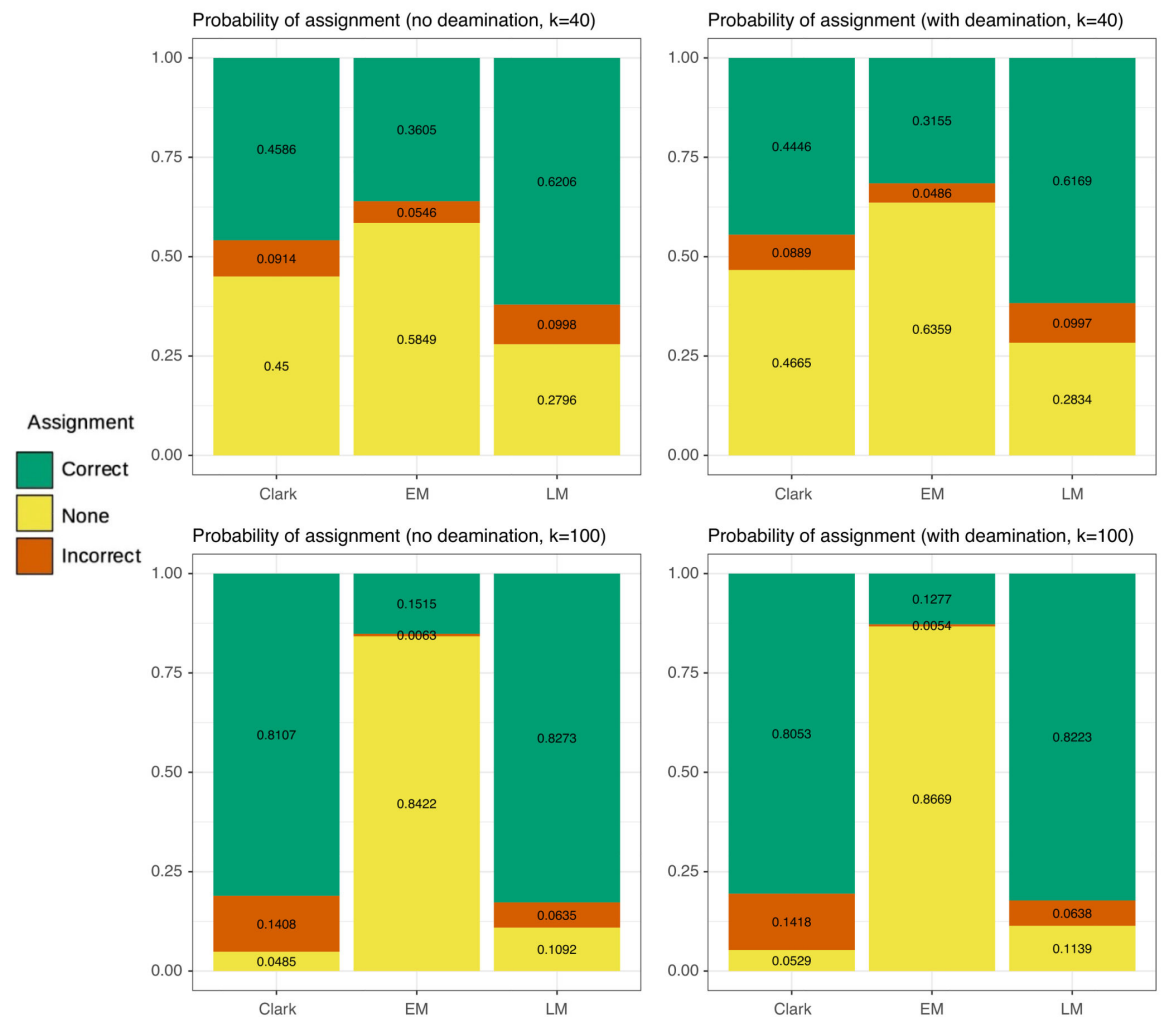
Simulated binning accuracy results using a parameter set motivated by a recent empirical study on ancient environmental DNA of black bears and giant short-faced bears. Results including deamination are shown on the right. Here, Clark refers to a discriminative k-mer-based method Clark, EM is ‘exact-match’, and LM is ‘least-mismatch’. Parameters used are given in the Methods

Results are shown in Figure 3. First of all, as expected, exact match is a more conservative method than least mismatch. This is especially true for a longer query sequence length, where the exactmatch method has a very low rate of incorrect assignments, but also assigns few total query sequences. This is because, when keeping other population genetic parameters constant, longer reads will be more likely to have mismatches with the reference sequence. Clark is an intermediate method between least mismatch and exact match in terms of both the total number of reads assigned and the proportion of reads which were assigned correctly. This is somewhat expected, since least mismatch effectively assigns reads wherever possible, and exact match is highly conservative in its assignments. However, when the read length is increased to 100, Clark has a higher rate of incorrect assignments than either exact match or least mismatch. We believe this is a consequence of Clark making decisions based on shorter k-mers than the whole sequence (we used the default k-mer size of 31, but the maximum k-mer length in Clark is 32). We expect this to be a general phenomenon of k-mer-based methods, including others such as Kraken (Wood et al., 2019), in scenarios with longer query reads that are expected to have fairly close reference sequences. We therefore do not recommend using k-mer-based methods in these situations. However, for very long query sequences such as scaffolds or chromosomes, those assembled with query reads, or when a nearby reference sequence does not exist, the full query may fail to align to any reference sequence and k-merbased methods may be superior.

We also simulated deamination, a damage process affecting ancient DNA, which introduces extra SNPs to the query reads. In theory, this will increase the number of mismatches between the query read and both the true and false sequences by the same number. Therefore, we expect that deamination should not impact the least mismatch method, but should reduce total assignments in both exact match and Clark. This is indeed what we see in Figure 3.

## 4 Conclusion

We hope that the theoretical and simulation framework presented here will add to an improved understanding of the uncertainties in the use of reference databases and binning methods when carrying out taxonomic assignment with both ancient and present-day metagenomic DNA. While many of the factors we discuss have already been acknowledged to be an issue in the community, there has previously been a lack of quantitative investigation of relationship of these parameters to the accuracy of taxonomic binning methods. We have also introduced a simulation framework to compare the least-mismatch and exact-match methods to a commonly used k-mer-based method Clark (Ounit et al., 2015) using parameters from a recent study to contextualize our results (Pedersen et al., 2021). We have made both the theoretical and simulation code publicly available, so that researchers may analyse and compare the performance of their own binning algorithms beyond fixed data sets or on specific parameters relating to their own studies. A framework in which to understand these issues is especially important as the fields of environmental and metagenomic DNA move into individual and population genetic analysis.

## Supplementary Material

Supplementary Table 1, Supplementary Figure 1

## Data Availability

Data sharing is not applicable to this article as no new data were created or analysed in this study.
