# Supplementary material for: A theoretical analysis of taxonomic binning accuracy
Source: Mol Ecol Resour. Author manuscript; Available in PMC 2025 Feb 24. (PMC7617423; doi:10.1111/1755-0998.13608)
Supplement: Supplementary Table 1, Supplementary Figure 1 [file EMS203358-supplement-Supplementary_Table_1__Supplementary_Figure_1.pdf]

Supplementary Information

Table 1: Comparison of theoretical and simulated results for values in Figure 1.

Assignment	Method	N	Ttf	k	Tqt	Theory	Simulated	p-value
correct	least-mismatch	4000	4e+05	96	0	0.5368	5407	0.4401
incorrect	least-mismatch	4000	4e+05	96	0	0.0036	44	0.1531
no	least-mismatch	4000	4e+05	96	0	0.4596	4549	0.3456
correct	exact-match	4000	4e+05	96	0	0.5314	5353	0.4404
incorrect	exact-match	4000	4e+05	96	0	0.0035	42	0.2334
no	exact-match	4000	4e+05	96	0	0.4651	4605	0.3617
correct	least-mismatch	6000	4e+05	96	0	0.5372	5347	0.6232
incorrect	least-mismatch	6000	4e+05	96	0	0.0053	49	0.6303
no	least-mismatch	6000	4e+05	96	0	0.4575	4604	0.5673
correct	exact-match	6000	4e+05	96	0	0.5292	5261	0.5412
incorrect	exact-match	6000	4e+05	96	0	0.0052	48	0.6754
no	exact-match	6000	4e+05	96	0	0.4657	4691	0.4955
correct	least-mismatch	8000	4e+05	96	0	0.5375	5406	0.5341
incorrect	least-mismatch	8000	4e+05	96	0	0.0071	70	0.9525
no	least-mismatch	8000	4e+05	96	0	0.4554	4524	0.5469
correct	exact-match	8000	4e+05	96	0	0.5269	5311	0.4002
incorrect	exact-match	8000	4e+05	96	0	0.0068	70	0.8076
no	exact-match	8000	4e+05	96	0	0.4663	4619	0.3832
correct	least-mismatch	10000	4e+05	96	0	0.5377	5363	0.7789
incorrect	least-mismatch	10000	4e+05	96	0	0.0089	69	0.0373
no	least-mismatch	10000	4e+05	96	0	0.4534	4568	0.501

correct	exact-match	10000	4e+05	96	0	0.5247	5246	0.992
incorrect	exact-match	10000	4e+05	96	0	0.0084	67	0.0625
no	exact-match	10000	4e+05	96	0	0.4669	4687	0.7258
correct	least-mismatch	12000	4e+05	96	0	0.538	5369	0.8332
incorrect	least-mismatch	12000	4e+05	96	0	0.0106	105	0.9611
no	least-mismatch	12000	4e+05	96	0	0.4514	4526	0.8095
correct	exact-match	12000	4e+05	96	0	0.5224	5239	0.7716
incorrect	exact-match	12000	4e+05	96	0	0.01	101	0.8801
no	exact-match	12000	4e+05	96	0	0.4676	4660	0.7561
correct	least-mismatch	14000	4e+05	96	0	0.5382	5433	0.3063
incorrect	least-mismatch	14000	4e+05	96	0	0.0124	124	1
no	least-mismatch	14000	4e+05	96	0	0.4494	4443	0.3053
correct	exact-match	14000	4e+05	96	0	0.5202	5259	0.2581
incorrect	exact-match	14000	4e+05	96	0	0.0115	119	0.7077
no	exact-match	14000	4e+05	96	0	0.4682	4622	0.2292
correct	least-mismatch	16000	4e+05	96	0	0.5384	5357	0.595
incorrect	least-mismatch	16000	4e+05	96	0	0.0142	132	0.4461
no	least-mismatch	16000	4e+05	96	0	0.4475	4511	0.4691
correct	exact-match	16000	4e+05	96	0	0.5181	5166	0.7717
incorrect	exact-match	16000	4e+05	96	0	0.013	118	0.3101
no	exact-match	16000	4e+05	96	0	0.4689	4716	0.5954
correct	least-mismatch	18000	4e+05	96	0	0.5385	5322	0.2063
incorrect	least-mismatch	18000	4e+05	96	0	0.0159	148	0.4014
no	least-mismatch	18000	4e+05	96	0	0.4455	4530	0.1339
correct	exact-match	18000	4e+05	96	0	0.5159	5110	0.3269
incorrect	exact-match	18000	4e+05	96	0	0.0145	134	0.3796

no	exact-match	18000	4e+05	96	0	0.4696	4756	0.2293
correct	least-mismatch	20000	4e+05	96	0	0.5387	5371	0.7559
incorrect	least-mismatch	20000	4e+05	96	0	0.0177	183	0.6218
no	least-mismatch	20000	4e+05	96	0	0.4436	4446	0.8484
correct	exact-match	20000	4e+05	96	0	0.5138	5153	0.7641
incorrect	exact-match	20000	4e+05	96	0	0.0159	161	0.873
no	exact-match	20000	4e+05	96	0	0.4703	4686	0.741
correct	least-mismatch	10000	2e+05	96	0	0.3262	3264	0.9745
incorrect	least-mismatch	10000	2e+05	96	0	0.0129	114	0.1842
no	least-mismatch	10000	2e+05	96	0	0.6608	6622	0.7675
correct	exact-match	10000	2e+05	96	0	0.3194	3210	0.7396
incorrect	exact-match	10000	2e+05	96	0	0.0124	111	0.2771
no	exact-match	10000	2e+05	96	0	0.6682	6679	0.9492
correct	least-mismatch	10000	6e+05	96	0	0.6828	6917	0.0558
incorrect	least-mismatch	10000	6e+05	96	0	0.0061	53	0.3351
no	least-mismatch	10000	6e+05	96	0	0.3111	3030	0.0802
correct	exact-match	10000	6e+05	96	0	0.6644	6748	0.0284
incorrect	exact-match	10000	6e+05	96	0	0.0057	50	0.3885
no	exact-match	10000	6e+05	96	0	0.3298	3202	0.0412
correct	least-mismatch	10000	8e+05	96	0	0.7823	7904	0.0511
incorrect	least-mismatch	10000	8e+05	96	0	0.0042	32	0.1403
no	least-mismatch	10000	8e+05	96	0	0.2135	2064	0.0853
correct	exact-match	10000	8e+05	96	0	0.7596	7698	0.0175
incorrect	exact-match	10000	8e+05	96	0	0.0039	31	0.2282
no	exact-match	10000	8e+05	96	0	0.2365	2271	0.0278
correct	least-mismatch	10000	1e+06	96	0	0.8507	8547	0.2618

incorrect	least-mismatch	10000	1e+06	96	0	0.0029	25	0.5741
no	least-mismatch	10000	1e+06	96	0	0.1465	1428	0.302
correct	exact-match	10000	1e+06	96	0	0.8245	8284	0.3115
incorrect	exact-match	10000	1e+06	96	0	0.0027	25	0.846
no	exact-match	10000	1e+06	96	0	0.1728	1691	0.3278
correct	least-mismatch	10000	4e+05	32	0	0.2293	2276	0.6947
incorrect	least-mismatch	10000	4e+05	32	0	0.0049	41	0.2535
no	least-mismatch	10000	4e+05	32	0	0.7656	7683	0.5316
correct	exact-match	10000	4e+05	32	0	0.2279	2260	0.6678
incorrect	exact-match	10000	4e+05	32	0	0.0049	41	0.3135
no	exact-match	10000	4e+05	32	0	0.7673	7699	0.5384
correct	least-mismatch	10000	4e+05	64	0	0.4042	3974	0.169
incorrect	least-mismatch	10000	4e+05	64	0	0.0076	80	0.6455
no	least-mismatch	10000	4e+05	64	0	0.5882	5946	0.1935
correct	exact-match	10000	4e+05	64	0	0.3981	3919	0.209
incorrect	exact-match	10000	4e+05	64	0	0.0074	77	0.6827
no	exact-match	10000	4e+05	64	0	0.5945	6004	0.2335
correct	least-mismatch	10000	4e+05	128	0	0.6401	6357	0.3648
incorrect	least-mismatch	10000	4e+05	128	0	0.0092	85	0.496
no	least-mismatch	10000	4e+05	128	0	0.3507	3558	0.2899
correct	exact-match	10000	4e+05	128	0	0.6182	6143	0.4281
incorrect	exact-match	10000	4e+05	128	0	0.0085	76	0.3548
no	exact-match	10000	4e+05	128	0	0.3733	3781	0.3261
correct	least-mismatch	10000	4e+05	160	0	0.7188	7200	0.7981
incorrect	least-mismatch	10000	4e+05	160	0	0.009	91	0.8737
no	least-mismatch	10000	4e+05	160	0	0.2722	2709	0.7788

correct	exact-match	10000	4e+05	160	0	0.6866	6879	0.7959
incorrect	exact-match	10000	4e+05	160	0	0.0081	83	0.8233
no	exact-match	10000	4e+05	160	0	0.3053	3038	0.7611
incorrect	exact-match	10000	4e+05	96	1e+05	0.0485	475	0.6417
no	exact-match	10000	4e+05	96	1e+05	0.5549	5614	0.1943
correct	least-mismatch	10000	4e+05	96	150000	0.4219	4156	0.2021
incorrect	least-mismatch	10000	4e+05	96	150000	0.0834	838	0.885
no	least-mismatch	10000	4e+05	96	150000	0.4947	5006	0.238
correct	exact-match	10000	4e+05	96	150000	0.3425	3395	0.5411
incorrect	exact-match	10000	4e+05	96	150000	0.0672	677	0.8417
no	exact-match	10000	4e+05	96	150000	0.5903	5928	0.6184
correct	least-mismatch	10000	4e+05	96	2e+05	0.3839	3813	0.6001
incorrect	least-mismatch	10000	4e+05	96	2e+05	0.1117	1113	0.9241
no	least-mismatch	10000	4e+05	96	2e+05	0.5044	5074	0.5552
correct	exact-match	10000	4e+05	96	2e+05	0.2942	2895	0.3127
incorrect	exact-match	10000	4e+05	96	2e+05	0.085	848	0.9571
no	exact-match	10000	4e+05	96	2e+05	0.6208	6257	0.3225
correct	least-mismatch	10000	4e+05	96	250000	0.3465	3444	0.6666
incorrect	least-mismatch	10000	4e+05	96	250000	0.1414	1391	0.5185
no	least-mismatch	10000	4e+05	96	250000	0.5121	5165	0.3787
correct	exact-match	10000	4e+05	96	250000	0.2511	2503	0.8536
incorrect	exact-match	10000	4e+05	96	250000	0.1019	1017	0.9605
no	exact-match	10000	4e+05	96	250000	0.6469	6480	0.8343
correct	least-mismatch	10000	4e+05	96	3e+05	0.3102	3114	0.787
incorrect	least-mismatch	10000	4e+05	96	3e+05	0.1723	1758	0.354
no	least-mismatch	10000	4e+05	96	3e+05	0.5176	5128	0.3418

correct	exact-match	10000	4e+05	96	3e+05	0.2131	2151	0.6253
incorrect	exact-match	10000	4e+05	96	3e+05	0.1179	1203	0.4568
no	exact-match	10000	4e+05	96	3e+05	0.669	6646	0.3553

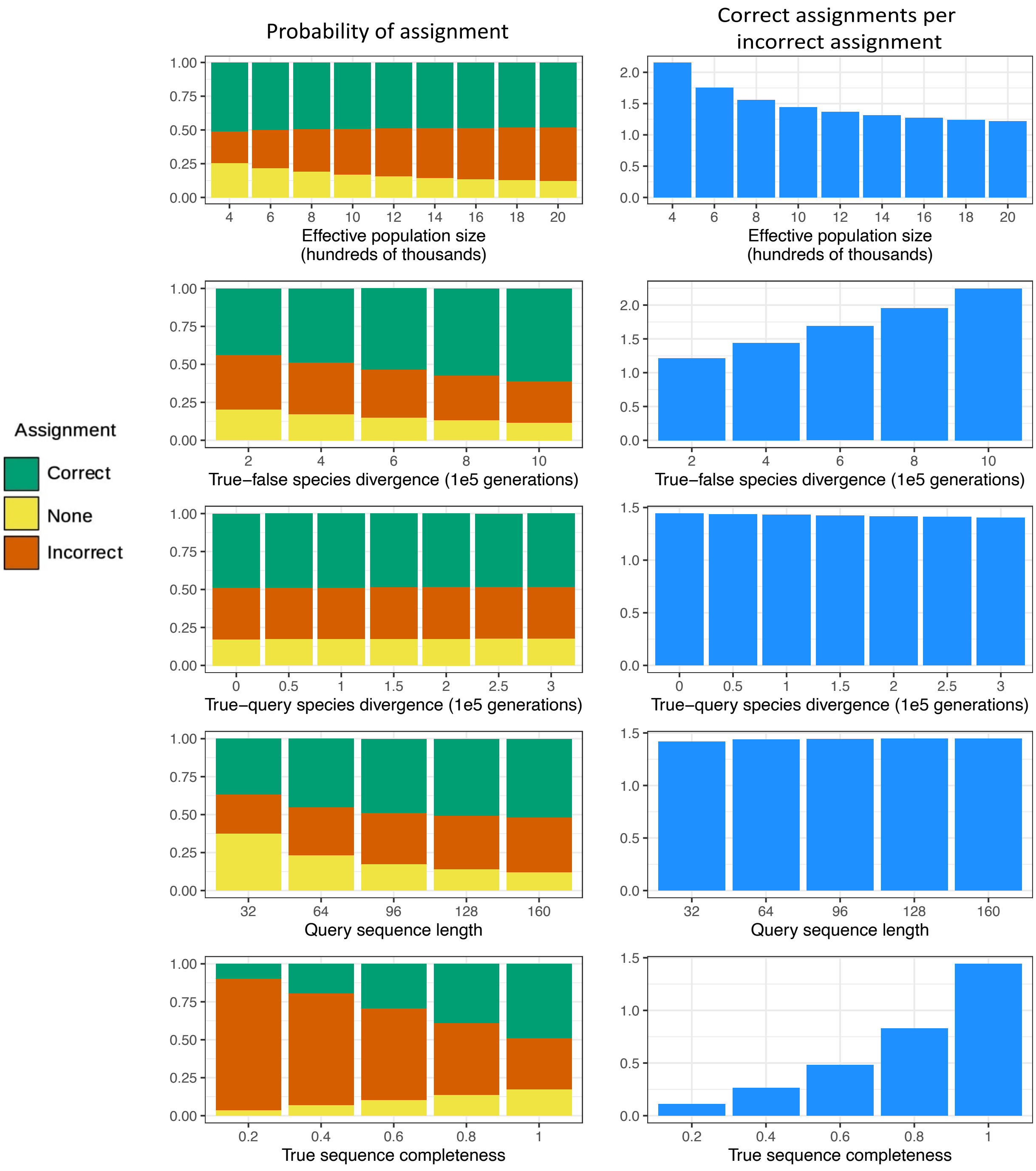

Supplementary Figure 1: A replicate of Figure 2, but with effective population sizes two orders of magnitude larger. Left: Probability of assigning the query sequence correctly (to the true reference sequence) in green, of assigning the query sequence incorrectly (to the false reference sequence) in red, and of making no assignment in yellow, using the least mismatch method. Right: The expected number of correct assignments (to the true reference sequence) made for every one incorrect assignment (to the false reference sequence). In both, each row varies a different parameter while keeping the others constant.
